# Detection of ESBL-producing *Escherichia coli* carrying *bla*_CTXM-15_ in magnificent frigatebird (*Fregata magnificens*) from Brazil: a one health perspective

**DOI:** 10.3389/fmicb.2025.1671302

**Published:** 2025-09-19

**Authors:** Bruno Rocha Pribul, Letícia da Silva Nascimento, Carlos Eduardo Gaspar Marinato, Melise Chaves Silveira, Daiana Cristina Silva Rodrigues, Bruna Ribeiro Sued-Karam, Daniel Miceli Serwy, Maria Ogrzewalska, Marina Galvão Bueno, Ana Paula D’Alincourt Carvalho-Assef, Miliane Moreira Soares de Souza, Cláudio Marcos Rocha-de-Souza

**Affiliations:** ^1^Laboratório de Bacteriologia Aplicada à Saúde Única e Resistência Antimicrobiana (LabSUR), Instituto Oswaldo Cruz (IOC), Fundação Oswaldo Cruz (Fiocruz), Rio de Janeiro, Brazil; ^2^Coleção de Culturas de bactérias de origem Hospitalar (CCBH), Instituto Oswaldo Cruz (IOC), Fundação Oswaldo Cruz (Fiocruz), Rio de Janeiro, Brazil; ^3^Laboratório de Bioinformática, Laboratório Nacional de Computação Científica (LNCC/MCTIC), Rio de Janeiro, Brazil; ^4^Laboratório de Vírus Respiratórios, Exantemáticos, Enterovírus e Emergências Virais, Instituto Oswaldo Cruz (IOC), Fundação Oswaldo Cruz (Fiocruz), Rio de Janeiro, Brazil; ^5^Laboratório de Virologia Comparada e Ambiental (LVCA), Instituto Oswaldo Cruz (IOC), Fundação Oswaldo Cruz (Fiocruz), Rio de Janeiro, Brazil; ^6^Departamento de Microbiologia e Imunologia Veterinária, Universidade Federal Rural do Rio de Janeiro (UFRRJ), Rio de Janeiro, Brazil

**Keywords:** antimicrobial resistance, ESBL-producing *Escherichia coli*, seabirds, *bla*_CTXM-15_, one health

## Abstract

**Background:**

Wild birds are increasingly recognised as sentinels for antimicrobial resistance (AMR) in environments impacted by human activity, yet the role of seabirds in the dissemination and maintenance of extended-spectrum *β*-lactamase (ESBL)-producing *Escherichia coli* in Brazil remains unclear.

**Methods:**

Cloacal swabs were collected from fifteen magnificent frigatebirds (*Fregata magnificens*) from the Cagarras Islands, a coastal archipelago. Bacterial isolation was performed using MacConkey agar supplemented with ceftriaxone, followed by identification using MALDI-TOF MS. Antimicrobial susceptibility testing was conducted using the disc diffusion method, and PCR screening was performed for ESBL genes. WGS and bioinformatics analysis were employed to characterise the isolate.

**Results:**

One ceftriaxone-resistant *E. coli* isolate was recovered from an adult female bird. The isolate was identified as sequence type ST5614 and serotype O27:H14, carrying the *bla*_CTX-M-15_ gene on an IncB/O/K/Z plasmid closely related to those described in human isolates. The strain showed resistance to multiple antimicrobials and harboured additional resistance genes including tet(A), sul1, sul2, mph(A), qnrS1, mrx(A), aph(3”)-Ib, aph(6)-Id, and ant(3”)-Ia.

**Conclusion:**

Detection of *bla*_CTX-M-15_ in *F. magnificens* may reflect the movement of clinically significant resistance genes at the human–wildlife interface, underscoring the value of seabirds as sentinels for environmental AMR surveillance. The findings highlight the interconnectedness of environmental, animal, and human health and reinforce the importance of wildlife surveillance in One Health AMR strategies.

## 1 Introduction

Multidrug-resistant (MDR) bacteria, defined as non-susceptibility to at least one agent in three or more antimicrobial categories (Magiorakos et al., 2012), have been detected in environmental sources such as rivers, sewage, and coastal waters. Wild birds are recognized as reservoirs and potential vectors of these pathogens, yet their role in the dissemination of antimicrobial resistance (AMR) remains poorly studied worldwide, including in Brazil (Sanganyado and Gwenzi, 2019; Paschoal et al., 2020). AMR is a major global health threat, exacerbated by improper disposal of antimicrobial-laden waste. Wild birds may acquire resistant strains from contaminated environments and contribute to their spread. The presence of MDR bacteria in birds without direct antimicrobial exposure highlights their potential as sentinels in AMR surveillance (Wang et al., 2017; Athanasakopoulou et al., 2022).

Extended spectrum *β*-lactamase (ESBL)-producing *Escherichia coli* (ESBL-EC) are a growing concern due to their resistance to critical antibiotics. While widely reported in hospitals, livestock, and companion animals, ESBL-EC have also been increasingly identified in wildlife and natural environments, suggesting broader ecological dissemination. In wild birds, especially those not exposed to antimicrobials, the detection of such resistant strains points to environmental acquisition and highlights the possible role of these animals as sentinels of antimicrobial resistance (Stedt et al., 2015; Fernandes et al., 2018; Silva et al., 2018; Salgado-Caxito et al., 2021).

The magnificent frigatebird (*Fregata magnificens*), a seabird widely distributed along tropical and subtropical coasts, nests on islands and disperses over large distances (Saviolli et al., 2016). Despite this, little is known about the occurrence of antimicrobial-resistant bacteria in its populations. Given its ecological role and exposure to anthropogenically influenced habitats, *F. magnificens* may help reveal resistance dynamics in coastal ecosystems (Zaluski et al., 2019). Expanding such investigations is crucial to support One Health strategies and address environmental dimensions of AMR.

This study investigated the presence of ESBL-producing *E. coli* in cloacal samples from 15 *F. magnificens* individuals inhabiting the Cagarras Islands, a coastal archipelago near Rio de Janeiro, Brazil. Microbiological analyses and whole genome sequencing were employed to characterize phenotypic and genotypic resistance profiles, providing new insights into the environmental dissemination of clinically relevant resistant bacteria and reinforcing the role of wildlife in One Health AMR surveillance strategies.

## 2 Materials and methods

### 2.1 Study area and sample collection

**Figure 1:**
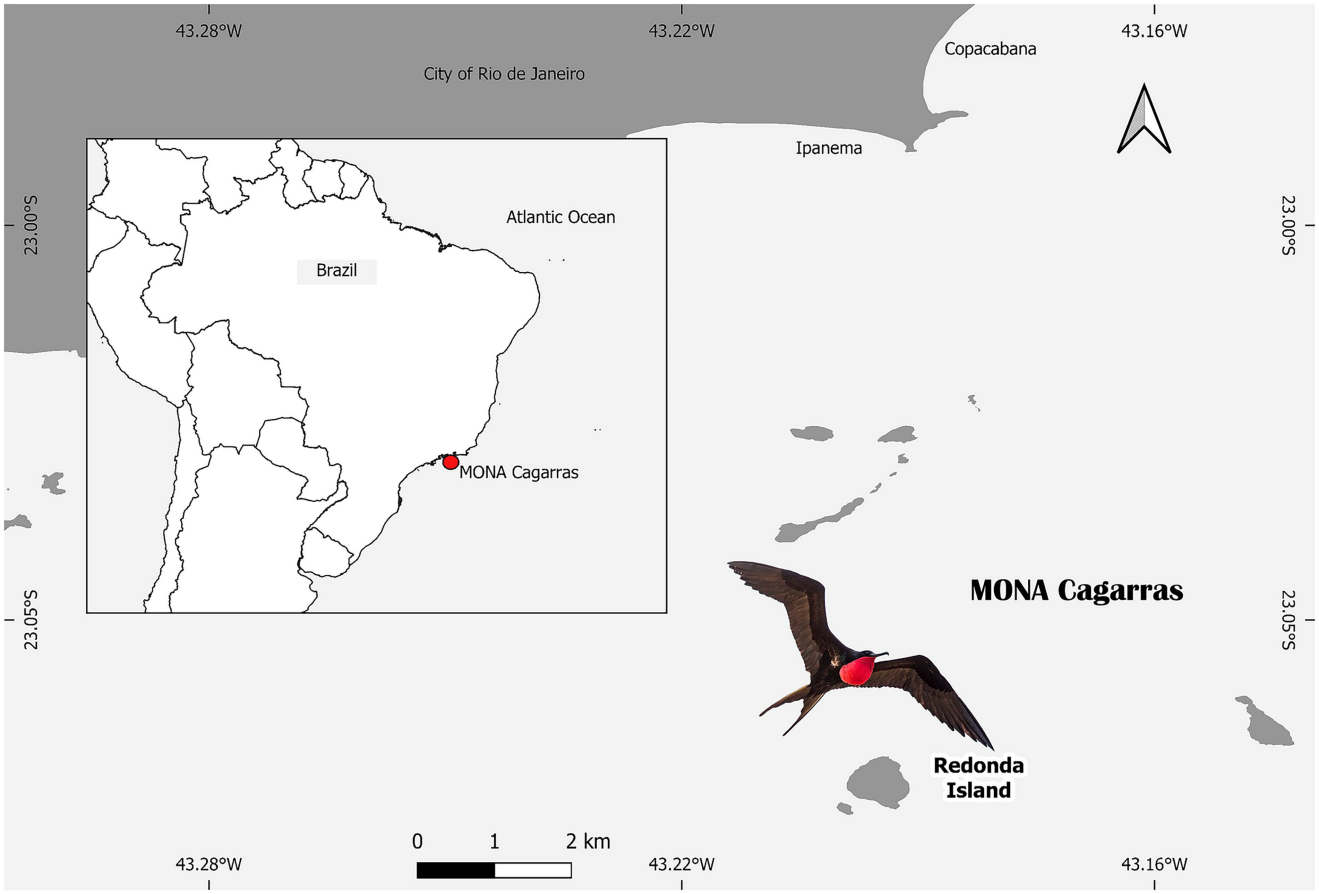
Map showing the location of the Cagarras Islands Natural Monument (MONA Cagarras) off the coast of Rio de Janeiro, Brazil, highlighting Redonda Island, where samples were collected. The inset indicates the position of MONA Cagarras along the southeastern Brazilian coast. The scale bar is provided, and Redonda Island is located approximately 5 km from the mainland at Ipanema Beach. The image also features a *Fregata magnificens*, a common seabird in the region (source: Generated by authors using QGIS 3.32.2 based on public governmental shapefiles).

The study was conducted in the Cagarras Islands Natural Monument (MONA Cagarras), located off the coast of Rio de Janeiro State, Brazil (23°01′S, 43°12′W), approximately 5 km from Ipanema Beach. The archipelago comprises six main landforms: the Cagarras, Palmas, Comprida, and Redonda islands, as well as the islets Filhote da Cagarras and Filhote da Redonda (Figure 1). Samples were collected in October 2023 on Redonda Island—the largest and highest landmass in the region, covering approximately 395,500 m^2^ and located 8.5 km from the mainland. This island is the main breeding and roosting site for *F. magnificens* and other marine bird species. Cloacal swabs were collected from each individual, placed in Cary Blair transport medium, kept refrigerated at 4 °C, and processed within 48 h. All sampling procedures were conducted under SISBIO permit no. 73163-6 and approved by the Animal Ethics Committee of the Oswaldo Cruz Institute (IOC), FIOCRUZ (Protocol L-019/2021).

### 2.2 Bacterial isolation and identification

Cloacal samples were streaked onto MacConkey agar plates supplemented with ceftriaxone (2 μg/mL) and incubated overnight at 35 ± 2 °C for 18–20 h to select for ESBL-EC strains. After incubation, a limited bacterial growth was observed. Colonies displaying growth under these selective conditions were subcultured in BHI broth containing ceftriaxone (2 μg/mL) for confirmatory screening. Following this selective enrichment process, only one cloacal sample yielded growth of a single bacterial species in pure culture. This isolate was identified by Matrix-Assisted Laser Desorption Ionization Time-of-Flight Mass Spectrometry (MALDI-TOF MS, Bruker Daltonik, Germany).

### 2.3 Antimicrobial susceptibility testing

Acquired antimicrobial resistance genes were detected with ResFinder (Bortolaia et al., 2020), and insertion sequences and transposable elements were detected with ISfinder (Siguier et al., 2006). Comparative plasmid analysis was performed using BLAST Ring Image Generator (BRIG) v0.95 (Alikhan et al., 2011). Antimicrobial susceptibility profiles were determined by the disc diffusion method using the following antimicrobial agents: sulbactam/ampicillin [SAM, 20 (10/10 μg)], tetracycline (TET, 30 μg), imipenem (IPM, 10 μg), meropenem (MEM, 10 μg), ciprofloxacin (CIP, 5 μg), amikacin (AK, 30 μg), gentamicin (CN, 10 μg), sulphamethoxazole/trimethoprim (SXT, 25 μg), clindamycin (DS, 2 μg), levofloxacin (LEV, 5 μg), nalidixic acid (NAL, 30 μg), tigecycline (TGC, 15 μg), ceftriaxone (CRO, 30 μg) and ceftazidime (CAZ, 30 μg). Phylogenetic tree was constructed using Parsnp v2.1.4 (Kille et al., 2024) based on the whole-genome alignment of *E. coli* 99RCEF. Publicly available genomes of E. coli isolates belonging to the same ST were retrieved from EnteroBase5 and included in the analysis. The resulting core genome alignment was used to infer a maximum likelihood phylogeny with IQ-TREE (Minh et al., 2020). employing the best-fit substitution model selected by ModelFinder (Kalyaanamoorthy et al., 2017) and performing 1,000 ultrafast bootstrap (Hoang et al., 2018) replicates to assess branch support. The final tree was visualized, edited, and annotated using Interactive Tree Of Life (iTOL) v76 (Letunic et al., 2024). Polymyxin B minimum inhibitory concentration (MIC) was determined by the broth microdilution method, in accordance with BrCast/EUCAST guidelines.^1^

### 2.4 PCR screening

Screening for ESBL genes was performed by PCR using specific primers for each target gene. The *bla*_CTX-M_ gene was amplified with primers Ctx-m-FW (5′-AAAAATCACTGCGCCAGTTC-3′) and Ctx-m-RV (5′-CCGTCGGTGACGATTTTAGCC-3′) (Saladin et al., 2002). For *bla*_TEM_, primers Tem-F (5′-ATGAGTATTCAACATT TCCGTG-3′) and Tem-R (5′-TTACCAATGCTTAATCAGTGAG-3′) were used, and for *bla*_SHV_, primers Shv-FW (5′-TTTATCGGCCCTCAC TCAAGG-3′) and Shv-RV (5′-GCTGCGGGCCGGATAACG-3′) were employed (Yuan et al., 2021). PCR cycling parameters (applied to all three assays): initial denaturation 94 °C for 3 min; 35 cycles of 94 °C for 45 s, 56 °C for 45 s, 72 °C for 45 s; final extension 72 °C for 10 min. As a positive control, the *Klebsiella pneumoniae* strain CCBH6556, harboring all three ESBL genes (GenBank: NZ_JBHFPY000000000.1), obtained from the Culture Collection of Hospital-Acquired Bacteria (CCBH), registered with the World Federation for Culture Collections (WFCC, WDCM 947), was used.

### 2.5 Whole genome sequencing and bioinformatics analysis

Genomic DNA was extracted using the QIAamp DNA Mini Kit (QIAGEN), according to the manufacturer’s protocol. Illumina libraries were prepared using the Illumina DNA Prep Kit and sequenced on a MiSeq platform (MiSeq Reagent Kit v2, 500 cycles). Raw Illumina reads were quality-filtered and adapter-trimmed using the CABGen web application (Duré et al., 2022). Oxford Nanopore Technologies (ONT) libraries were constructed with the Rapid Barcoding Kit 24 V14 (SQK-RBK114.24, ONT) and sequenced on a MinION Mk1B with an R10.4.1 flow cell, operated via MinKNOW software. Basecalling of ONT signal data was performed using Dorado.^2^

Hybrid *de novo* assembly was performed with Unicycler v0.4.9 (conservative mode) (Wick et al., 2017). Assembly completeness and contamination were evaluated using CheckM (Parks et al., 2015), and taxonomic classification and contaminant screening were performed with Kraken2 (Wood et al., 2019). Genome annotation was carried out using Prokka (Seemann, 2014). Plasmid replicon typing was performed with PlasmidFinder (Carattoli et al., 2014), serotyping with SerotypeFinder (Joensen et al., 2015), and multilocus sequence typing (MLST) using the Achtman scheme.^3^ Acquired antimicrobial resistance genes were detected with ResFinder (Bortolaia et al., 2020), and insertion sequences and transposable elements were detected with ISfinder.^4^ Comparative plasmid analysis was performed using BLAST Ring Image Generator (BRIG) v0.95.

Phylogenetic tree was constructed using Parsnp v2.1.4 (10.1093/bioinformatics/btae311) based on the whole-genome alignment of *E. coli* 99RCEF. Publicly available genomes of *E. coli* isolates belonging to the same ST were retrieved from EnteroBase^5^ and included in the analysis. The resulting core genome alignment was used to infer a maximum likelihood phylogeny with IQ-TREE (v2.2.010.1093/molbev/msaa015), employing the best-fit substitution model selected by ModelFinder and performing 1,000 ultrafast bootstrap replicates to assess branch support. The final tree was visualized, edited, and annotated using Interactive Tree Of Life (iTOL) v7.^6^

## 3 Results

A total of 15 *F. magnificens* individuals (10 adults and five juveniles; comprising two males, eight females, and five juveniles of undetermined sex) were captured on Redonda Island, Cagarras Archipelago. Cloacal swabs were obtained from all apparently healthy birds. Among the 15 samples, one ceftriaxone-resistant *E. coli* isolate was recovered from an adult female bird (6.6%; 95% CI: 0.16%–31.95%). Identification by MALDI-TOF MS confirmed the isolate as *E. coli*, and PCR detected the presence of the *bla*_CTXM_ gene.

The antimicrobial susceptibility profile revealed that the isolate was resistant to tetracycline, nalidixic acid, trimethoprim-sulfamethoxazole, clindamycin, ciprofloxacin, ceftriaxone and ceftazidime.

**Figure 2:**
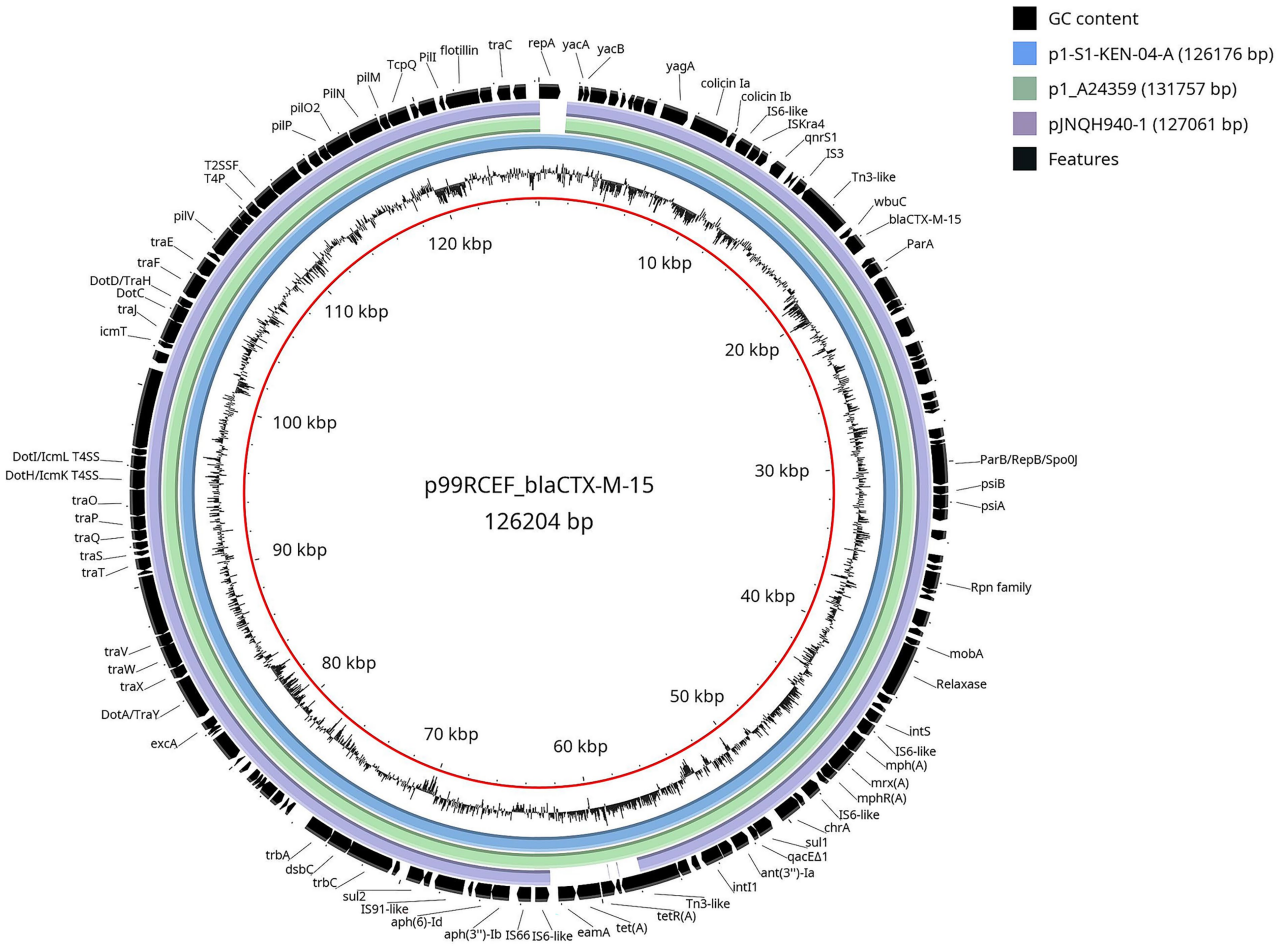
Genomic comparison between IncB/O/K/Z plasmids in relation to p99RCEF_blaCTX-M-15 from *E. coli* 99RCEF. Inside out, the first ring is plasmid p99RCEF_blaCTX-M-15 from *E. coli* 99RCEF. The second ring is the plasmid GC content. The outer rings represent the nucleotide sequence of the corresponding DNA region of the following IncB/O/K/Z plasmids in different colors against the reference genome (p99RCEF_blaCTX-M-15): p1-S1-KEN-04-A from *E. coli* (blue), p1_A24359 from *E. coli* (green), and pJNQH940-1 from *Salmonella enterica* (purple). The last one is the gene annotations (black).

Whole genome sequencing identified the strain as sequence type ST5614 and serotype O27:H14. The analysis also revealed the presence of a 126,204 bp plasmid, named p99RCEF_blaCTX-M-15 (Figure 2), classified within the IncB/O/K/Z incompatibility group. This plasmid harbored the *bla*_CTXM-15_ gene, as well as *tet(A)*, *sul1*, *sul2*, *mph(A)*, *qnrS1*, *mrx(A)*, *aph(3″)-Ib*, *aph(6)-Id*, and *ant(3″)-Ia*.

The *bla*_CTXM-15_ gene was located within a Tn2-like element flanked by intact inverted repeats, although the element lacked a complete transposition module. The plasmid encoded a full set of conjugal transfer genes (t*raA* to *traY*), toxin-antitoxin systems, pilus assembly proteins, colicin operons, recombinases, and components of a type IV secretion system.

Comparative genomic analysis showed that p99RCEF_blaCTX-M-15 shares high nucleotide sequence identity and structural similarity with three previously characterized IncB/O/K/Z plasmids carrying *bla*_CTXM-15_: p1-S1-KEN-04-A (126,176 bp; GenBank NZ_CP145691.1) and p1_A24359 (131,757 bp; GenBank CP183674.1), both isolated from *E. coli* strains recovered from human faecal material in Germany and Switzerland, respectively, and pJNQH940-1 (127,061 bp; GenBank NZ_CP136142.1) from *Salmonella enterica* isolated from a renal transplant patient in China. These plasmids share a conserved genetic backbone, including mobile genetic elements and regions conferring antimicrobial resistance.

**Figure 3:**
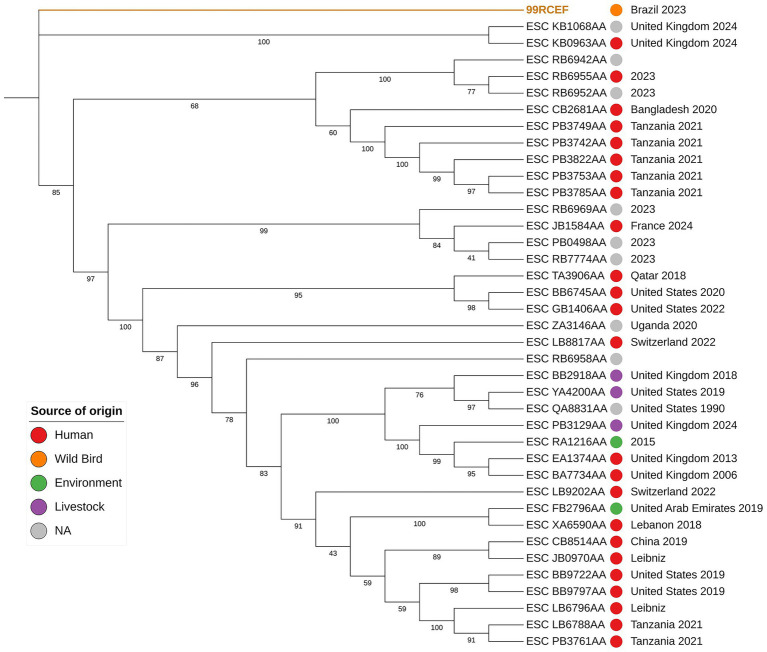
Maximum-likelihood phylogenetic tree based on core-genome single nucleotide polymorphisms (SNPs) from complete genomes of *E. coli* ST 5614, using 99RCEF as reference. The analysis includes all isolates of ST 5614 deposited in EnteroBase. Bootstrap values >40 are shown at the nodes. The colored circles indicate source of origin: red for human, orange for wild bird, green from environment, purple for livestock and gray for isolates with no available source information. Country and year of isolation are shown to the right of each isolate ID, when available in EnteroBase.

The isolate 99RCEF was identified as a phylogenetically distinct and basal lineage within the maximum likelihood phylogenetic tree compared to all other *E. coli* isolates of the same sequence type included in this study (Figure 3). While most isolates, predominantly derived from human, livestock or environmental sources, formed multiple well supported and genetically diverse clades, 99RCEF branched independently from the rest of the dataset. This separation was marked by a considerably long branch length and a strongly supported internal node with a bootstrap value of 100, providing robust statistical evidence for the phylogenetic distinctiveness and divergence of 99RCEF within the sequence type examined.

## 4 Discussion

The detection of an ESBL-EC strain carrying *bla*_CTXM-15_ in *F. magnificens* from the Cagarras Islands represents a significant finding in the context of environmental AMR surveillance in Brazil. Wild birds, particularly those inhabiting environments impacted by human activities, have emerged as important sentinels for monitoring the spread of AMR, yet their role in the dissemination and maintenance of resistant bacteria remains underexplored in the country. The MDR bacteria in environmental matrices such as rivers, sewage, and coastal waters underscores the growing ecological dimension of AMR and the need for integrated One Health approaches.

Our study revealed the presence of an ESBL-EC strain resistant to multiple antimicrobials critically important in both animal production and human medicine, in a seabird species with no direct exposure to antibiotics. Furthermore, findings from protected or uninhabited islands show clinically relevant AMR in seabirds without clinical treatment, consistent with indirect anthropogenic contamination and environmental selection rather than direct medicinal use (Ewbank et al., 2021c). This finding aligns with previous reports from Brazil and other countries, where wild birds have been shown to harbor MDR bacteria, often with resistance profiles similar to those found in clinical and livestock settings (Costa et al., 2006; Pinto et al., 2015; Silva et al., 2020; Martín-Maldonado et al., 2022).

The acquisition of such bacteria by wild birds is likely linked to environmental contamination, particularly in areas receiving untreated domestic and hospital sewage, as is the case for Guanabara Bay adjacent to the Cagarras Islands. Recent environmental studies in Guanabara Bay and recreational beaches near the Cagarras Archipelago have already reported the presence of multidrug-resistant bacteria and resistance genes, including *bla*_CTXM_ variants in water and sediment samples (Montezzi et al., 2015; de Araujo et al., 2016; Costa et al., 2023). These findings highlight the chronic impact of untreated domestic and hospital sewage on the region, creating hotspots for the selection and persistence of antimicrobial resistance determinants. The proximity of the Cagarras Islands to these impacted coastal areas, together with the opportunistic foraging behavior of *F. magnificens*, which includes scavenging on fishing discards, kleptoparasitism, and feeding near coastal human-impacted habitats, strongly suggests that these seabirds may acquire resistant bacteria from such contaminated environments (Schunck et al., 2025). This environmental context supports our findings and emphasizes the role of anthropogenic pollution in shaping the resistome of wildlife in the region.

The detection of a *bla*_CTXM-15_-positive IncB/O/K/Z plasmid in *E. coli* from *F. magnificens* aligns with recent evidence of the same plasmid type mediating multidrug resistance in clinical *S. enterica* isolate from blood of a hospitalised renal transplant patient in China (Ma et al., 2024). The strong genetic similarity between the p99RCEF_blaCTX-M-15 and clinical plasmids underscores the potential for interspecies and inter-environmental spread of critical resistance determinants, reinforcing the importance of One Health surveillance strategies.

The detection of an IncB/O/K/Z plasmid in *F. magnificens* contrasts with the predominance of IncF and IncI1 plasmids typically reported in seabirds (Wang et al., 2017; Athanasakopoulou et al., 2022). Furthermore, the identification of serogroup O27:H14 and ST5614 diverges from the more frequently observed serogroups in seabirds, such as O25b (ST131), O78 (ST117), and those associated with ST1159 and ST602 (Ewbank et al., 2022a; Dalazen et al., 2023). Notably, while serogroup O27 has been identified as the predominant serotype (75% of isolates) among Shiga toxin-producing *E. coli* strains from wild mammals including wild boar, red deer, otter, and fox in Portugal (Dias et al., 2022), its detection in marine birds appears to be previously unreported in the literature. The absence of documented O27 detection in Brazilian coastal waters or clinical surveillance studies further emphasizes the novelty of this finding and may suggest potential gaps in current epidemiological monitoring of *E. coli* serogroup diversity in marine environments. The detection of O27 *E. coli* in wild mammals coupled with its absence in current marine bird surveillance suggests that *F. magnificens* may serve as a carrier for this serogroup in this coastal environment.

The phylogenetic divergence of the 99RCEF isolate from other *E. coli* strains of the same ST may reflects a unique evolutionary history shaped by its host’s specific ecological context. Originating from a magnificent frigatebird inhabiting relatively isolated and anthropogenically impacted Cagarras islands, the founder population from which 99RCCEF belonged may have been subject to evolutionary forces such as genetic drift, positive selection and horizontal gene transfer (HGT) (Zdziarski et al., 2010; Martinez, 2012). These evolutionary processes may have influenced the development of its distinctive core phylogenetic markers, likely as an adaptive response to local antimicrobial selective pressures and interspecific competition within the microbial community. The ecological mobility and foraging behavior of *F. magnificens* likely facilitated contact with diverse microbial populations across different environments. This exposure to varied microbial communities, combined with local selective pressures, may collectively explain the genetic distinctiveness observed in isolate 99RCEF when compared to other strains of the same sequence type.

The epidemiological significance of detecting a plasmid with high structural similarity to those circulating in human and animal populations is considerable. Plasmids of the IncB/O/K/Z group are recognized as important vehicles for the global dissemination of *bla*_CTX-M-15_ and other resistance genes (Rozwandowicz et al., 2018; Shirakawa et al., 2020; Ma et al., 2024). The occurrence of such plasmids in a wild seabird from a region heavily impacted by sewage discharge suggests that environmental interfaces play a crucial role in the maintenance and spread of AMR determinants (Coutinho et al., 2013).

Wildlife, including seabirds such as *F. magnificens*, may act as both carriers and vectors of clinically relevant resistance genes, facilitating their movement between environmental, animal, and human populations. The foraging behavior of *F. magnificens*, which includes feeding on fish and fishery waste in areas such as Guanabara Bay, increases their exposure to anthropogenically derived contaminants, including MDR bacteria. Our findings represent the first identification of the *bla*_CTXM-15_ gene variant in *F. magnificens* from the Cagarras Islands. Previous research in the Alcatrazes Archipelago identified *E. coli* isolates harboring *bla*_CTX-M-2_ and *bla*_CMY-2_ genes in *F. magnificens* but did not detect *bla*_CTXM-15_ (Ewbank et al., 2022b).

The detection of *bla*_CTX-M_ variants in birds from both archipelagos, despite their geographic separation, reinforces the idea that wild seabirds in Brazil are consistently exposed to antimicrobial-resistant bacteria of clinical relevance. Considering the strong flight capacity and foraging range of *F. magnificens*, it is plausible that individuals may transit between these and other coastal areas, either directly or through overlapping feeding grounds. *Fregata magnificens* breeds on islands across the Caribbean and tropical coasts of Central and South America (Nuss et al., 2016). While some individuals remain near colonies due to the prolonged breeding season, others, especially non-breeders, disperse widely. GPS data have recorded post-breeding movements up to 1,400 km (Weimerskirch et al., 2006), such mobility could contribute to the regional circulation of resistant bacteria and genetic elements across marine ecosystems influenced by anthropogenic pollution.

The presence of *bla*_CTXM-15_ in wild birds has been reported worldwide, including Brazil (Guenther et al., 2012; Ben Yahia et al., 2018; Batalha de Jesus et al., 2019; Zurfluh et al., 2019; Beleza et al., 2024). These findings collectively may indicate the widespread environmental circulation of this clinically relevant resistance determinant. Our results provides important new insights into the genetic diversity and geographic spread of ESBL genes among marine avifauna along the Brazilian coastline by confirming the occurrence of *bla*_CTX-M-15_ in the *F. magnificens* population, and the identification of the IncB/O/K/Z plasmid p99RCEF_blaCTX-M-15 adds to the understanding of the diversity and distribution of resistance elements in wildlife.

In summary, the first detection of ESBL-EC carrying *bla*_CTX-M-15_ and a multidrug-resistant IncB/O/K/Z plasmid in *F. magnificens* from the Cagarras Islands underscores the interconnectedness of environmental, animal, and human health. While our sample size was constrained by the logistical challenges of accessing this protected marine environment, the study’s principal contribution lies in documenting an ecological bridge for a clinically important resistance gene rather than estimating prevalence. The genomic evidence demonstrates mechanistic connectivity that transcends sample size limitations, with the plasmid’s close structural relationship to clinical isolates providing clear evidence of cross-environmental exchange. These findings reinforce the importance of wildlife surveillance in One Health AMR strategies and highlight the need for continued monitoring of resistance dynamics in coastal ecosystems impacted by human activities.

## Data Availability

The datasets presented in this study can be found in online repositories. The names of the repository/repositories and accession number(s) can be found below: https://www.ncbi.nlm.nih.gov/, PRJNA1281631, https://www.ncbi.nlm.nih.gov/, SAMN49569905, https://www.ncbi.nlm.nih.gov/genbank/, NZ_CP196680.1.

## Footnotes

^1^ http://www.eucast.org

^2^ https://github.com/nanoporetech/dorado

^3^ https://pubmlst.org/organisms/escherichia-spp

^4^ https://isfinder.biotoul.fr/blast.php

^5^ https://enterobase.warwick.ac.uk/species/index/ecoli

^6^ https://itol.embl.de/
